# An Excellent Response of Microsatellite Instability-High Pancreatic Adenocarcinoma to Pembrolizumab Treatment: The Role of Circulating Tumor DNA Testing

**DOI:** 10.7759/cureus.37239

**Published:** 2023-04-07

**Authors:** Bhrugun Anisetti, Tucker W Coston, Ahmed K Ahmed, Himil J Mahadevia, Mark A Edgar, Jason S Starr, Hani M Babiker

**Affiliations:** 1 Division of Hematology and Medical Oncology, Mayo Clinic, Jacksonville, USA; 2 Department of Internal Medicine, University of Missouri Kansas City, Kansas City, USA; 3 Department of Laboratory Medicine and Pathology, Mayo Clinic, Jacksonville, USA

**Keywords:** microsatellite instability high, cytotoxic chemotherapy, pembrolizumab, ctdna testing, pancreatic adenocarcinoma

## Abstract

The role of circulating tumor DNA (ctDNA) is expanding in oncology practices, and it is increasingly being used for targeted therapies and disease monitoring. It is minimally invasive and provides data from both primary and secondary sites of disease. Herein, we report a unique case of a patient with microsatellite instability-high (MSI-H) pancreatic adenocarcinoma (PDAC) treated with neoadjuvant chemotherapy and pembrolizumab who achieved a pathologically confirmed complete resolution of the tumor.

A 75-year-old female was diagnosed with pancreatic adenocarcinoma (PDAC) in the uncinate process with aortocaval and retrocrural adenopathy. Next-generation sequencing was obtained via ctDNA testing, and the patient was initiated on cytotoxic chemotherapy while awaiting results. ctDNA revealed MSI-H status, and pembrolizumab was added to the cytotoxic chemotherapy regimen. At follow-up after five cycles of treatment, excellent treatment response was noted on magnetic resonance imaging (MRI) of the abdomen, demonstrating the resolution of the pancreatic mass and adenopathy. Six months of neoadjuvant treatment was given in total, after which the patient underwent resection with curative intent and achieved a complete pathological response with no evidence of disease.

The role of ctDNA testing in directing treatment and influencing follow-up has already demonstrated great value. In our case, ctDNA adequately replaced conventional tissue biopsy, alleviating the burden of invasive testing on the patient. This is of great value, especially for patients with non-resectable tumors as well as in several other clinical scenarios. Our case also contributes to the growing body of literature demonstrating the role of immune-directed therapy for MSI-H PDAC.

## Introduction

The role of circulating tumor DNA (ctDNA) testing is expanding, increasingly being used to identify biomarkers that guide clinical decision-making, which streamlines targeted treatment choices and disease monitoring. ctDNA, also termed “liquid biopsy” [1], is a minimally invasive approach that presents tumor genomic data from both primary and secondary tumor sites of disease [2].

Microsatellite instability (MSI) is a genomic signature associated with mutations leading to deficient DNA mismatch repair (dMMR), as seen in tumors occurring in Lynch syndrome (LS) [3]. MSI status is categorized as absent (termed as microsatellite stable or MSS), low (MSI-L), or high (MSI-H). The identification of MSI-H/dMMR as a biomarker for response to immune checkpoint blockade has been a breakthrough in the treatment of many advanced solid tumors [4,5]. Pembrolizumab, an inhibitor of programmed cell death protein 1 (PD-1) active in upregulating the antitumor activity of T-cells, is the first tumor-agnostic approval by the US FDA for the treatment of advanced MSI-H/dMMR solid tumors [6].

Here, we report a case wherein the findings imply that detecting MSI-H using a well-validated ctDNA could predict a robust response to immune checkpoint inhibitors in locally advanced pancreatic adenocarcinoma (PDAC), further highlighting the importance of ctDNA in guiding the patient’s treatment decisions and improved patient outcome.

## Case presentation

**Figure 1:**
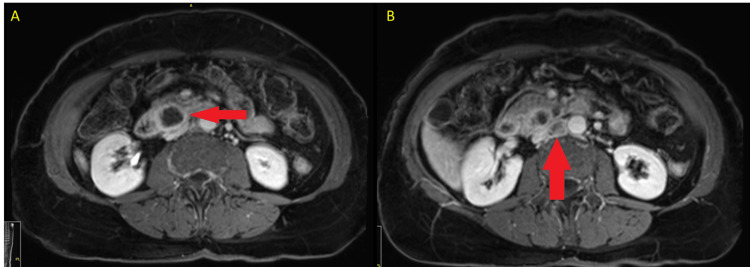
Initial MRI Axial post-contrast T1W MRI showing the (A) primary tumor, peripherally enhancing mass in the pancreatic uncinate process measuring up to 4.2 cm with an adjacent satellite lesion (2.3 cm), and (B) metastatic, necrotic inter-aortocaval lymph node measuring 1.5 × 2.0 × 4.6 cm. Both are highlighted with red arrows for clarity. MRI: magnetic resonance imaging

A 75-year-old female presented for oncology evaluation after a workup for progressive abdominal pain revealed a hetero-dense mass at the uncinate process of the pancreas without vascular involvement measuring 2.7 × 2.4 × 4.2 cm on cross-sectional imaging (Figure 1); necrotic retrocrural and aortocaval lymphadenopathy was also noted up to 2.5 cm. Fluorodeoxyglucose (FDG)-positron emission tomography (PET) demonstrated hyper-avidity of the primary tumor and the concerning lymph nodes. A pathological sampling of the primary pancreatic mass was obtained by esophagogastroduodenoscopy (EGD) with endoscopic ultrasound (EUS) and fine-needle aspiration (FNA), which confirmed the diagnosis of adenocarcinoma of pancreatic origin. CA19-9 shortly after diagnosis was within normal limits (9 units/mL) (normal range: 0-37 units/mL). The case was reviewed by our multidisciplinary hepatobiliary tumor board, and it was decided to initiate cytotoxic chemotherapy (gemcitabine 1,000 mg/m^2^ plus nab-paclitaxel 125 mg/m^2^, initially dosed days 1, 8, and 15 out of 28-day cycle but subsequently dosed every other week) with the intent to deliver six months of neoadjuvant therapy with subsequent en bloc resection. Shortly after the initiation of systemic therapy, tumor genomic sequencing was recommended. To check for alterations in MSI-H or *NTRK* fusions or other targetable mutations. Using a wide-ranging targeted gene panel (Guardant360, Guardant Health, Inc., CA, USA), which sequences 83 cancer-associated genes in human cancers, genomic alterations such as mutations, insertions, deletions, and amplifications were tested on the patient’s plasma ctDNA. The ctDNA was obtained through hybrid capture, and the analysis was performed on an Illumina platform and hg19 as the reference genome. The patient’s tumor was found to harbor KRAS G12D and NF1 R461 mutations but was most notable for demonstrating MSI-H status.

A subsequent discussion with the patient regarding her tumor’s MSI-H status, the high objective response rate to immunotherapy of other MSI-H solid tumors, and the potential risks versus benefits of treatment ultimately led to the addition of pembrolizumab 200 mg IV every three weeks to her standard cytotoxic chemotherapy regimen. The patient had received one cycle (three weekly doses of gemcitabine plus nab-paclitaxel) prior to the supplementation of the regimen with immunotherapy.

**Figure 2:**
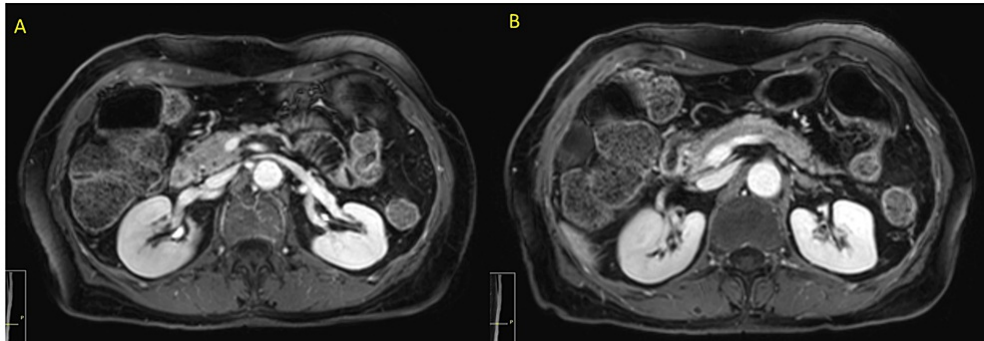
Restaging MRI Restaging MRI of the primary tumor site (A) and the prior site of the metastatic lymph node (B) demonstrating the best response to treatment. Radiographic CR was achieved with regression of the primary tumor and disappearance of the concerning lymph node. CR was later confirmed by surgical pathological evaluation. MRI: magnetic resonance imaging, CR: complete response

Early restaging imaging after two cycles of a single dose of pembrolizumab demonstrated a treatment response, with decreased size of the primary tumor (from 2.7 × 2.4 × 4.2 cm to 0.8 × 0.6 × 1.2 cm) as well as decreased size of the lymphadenopathy (from 1.5 × 2.0 × 4.6 cm to 1.3 × 0.7 × 1.4 cm). Subsequent restaging magnetic resonance imaging after six months of neoadjuvant chemo-immunotherapy demonstrated a radiographic complete response, with the resolution of the primary pancreatic mass and normalization of the lymphadenopathy (Figure 2).

**Figure 3:**
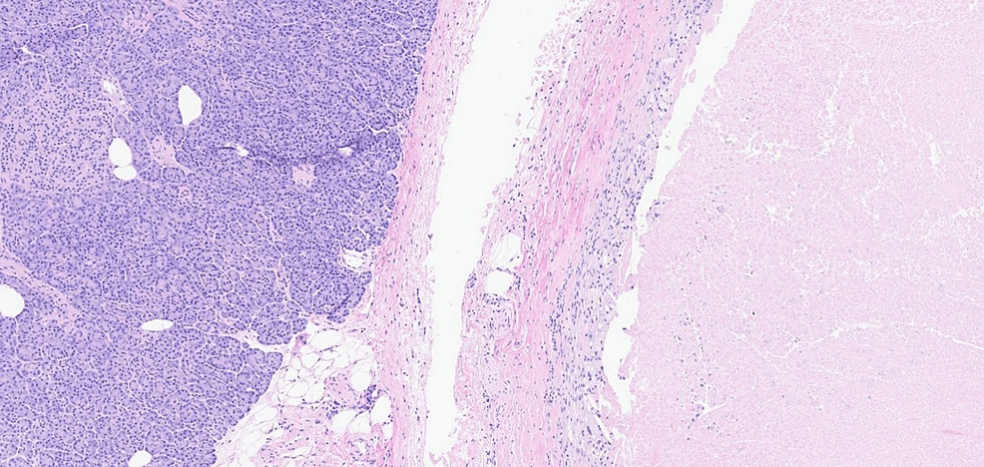
Histopathology slide: H&E stain A circumscribed focus of coagulative necrosis is rimmed by histiocytes on the right with a normal pancreas on the left (hematoxylin and eosin, 7.9×) H&E: hematoxylin and eosin

Planned pylorus-preserving pancreaticoduodenectomy with lymphadenectomy and complex reconstruction was completed by our surgical team, without immediate or postoperative complications. Surgical pathology demonstrated foci of organizing necrosis alone, without viable malignant cells seen in the primary tumor bed, the 29 regional lymph nodes excised, or any of the six other excised lymph nodes (retroperitoneal and porta hepatis). Incidentally noted in the surgical tissue was a focus of low-grade pancreatic intraepithelial neoplasia, a noninvasive precursor lesion to invasive pancreatic adenocarcinoma (Figure 3).

**Figure 4:**
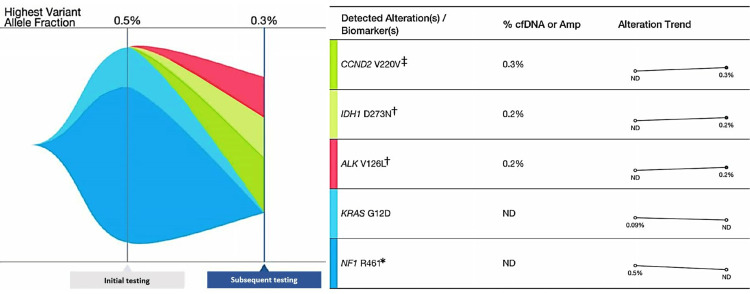
Tumor response map from ctDNA testing Tumor response map obtained from ctDNA testing, both initially and 18 months later. It shows the disappearance of the KRAS G12D and NF1 R461 mutations 18 months later. It also shows the detection of two variants of uncertain clinical significance (IDH D273N and ALK V126L) and a synonymous alteration (CCND V220V). ctDNA: circulating tumor DNA, ND: not detected, (‡): synonymous alteration, (†): uncertain clinical significance, (*): nonsense mutation

Postoperatively, the pathology results were reviewed in detail with the patient, along with the paucity of randomized clinical data to guide treatment in her situation. In a process of shared decision-making, it was decided to continue with pembrolizumab alone in the adjuvant setting to complete a year of immune-directed treatment, given the patient’s excellent tolerability and disease response. She has resumed immunotherapy without the development of adverse effects to date. Eighteen months after initial testing, follow-up ctDNA testing did not identify the KRAS G12D and NF1 R461 mutations or the MSI-H. Instead, it detected the presence of two variants, IDH D273N and ALK V126L, which have uncertain clinical significance. Additionally, a synonymous alteration, i.e., does not alter the amino acid sequence (CCND V220V), was also detected (Figure 4).

## Discussion

Our case highlights several benefits to be gained by utilizing ctDNA testing, as well as emphasizing the impressive therapeutic potential of immune-directed therapy in MSI-H PDAC. When testing genomic sequencing by conventional tissue biopsy, results may be subject to the incomplete characterization of the mutational profile if there is intratumoral genomic heterogeneity or genomic heterogeneity between the patient’s primary tumor and metastatic disease, albeit rare in PDAC [7]. More commonly, there is insufficient tumor tissue sampled via EUS and FNA to sequence the tumor genome. Genomic sequencing by ctDNA testing avoids these potential pitfalls by sampling the mutational profile across the entirety of the patient’s disease burden [8,9]. Furthermore, such an approach can be instrumental in patients in whom biopsy is not a feasible option, whether because of difficult anatomy, prohibitive procedural risk, or patient preference to avoid more invasive sampling. Given the accessibility and negligible risk of ctDNA testing, this allows for the possibility of serial genomic sequencing to monitor the development of resistance mechanisms to treatment, a strategy that offers the potential to guide therapy decisions based on these findings and will have broader implications in the future. In addition, ctDNA has shown promise as a reliable residual disease biomarker after neoadjuvant chemotherapy in nonmetastatic breast cancer, and trials are currently ongoing in colorectal cancer (CRC) that will guide adjuvant therapy in the future [10]. Recent literature has shown that ctDNA obtained through plasma, blood, and serum is a carrier of actual genetic composition in real-time and is potentially more informative than tissue-derived nucleic acids archived at the time of the diagnosis [11,12]. Moreover, genomic sequencing has proven beneficial in finding therapeutic targets for patients who progress after several lines of therapy that also includes early clinical trials matching based on therapeutic targets [13,14]. However, one drawback of the ctDNA testing method used in this patient is that it is unable to determine the source of the ctDNA. As a result, a combined radiological assessment was necessary to pinpoint the location of the tumor.

As discussed above, MMR deficiency (as is seen in cells with mutated *MLH1*, *MSH2*, *MSH6*, and *PMS2* genes) leads to high MSI, both of which are markers of response to immunotherapy in many tumor types. LS is a hereditary cancer syndrome defined by a germline pathogenic mutation of any of the DNA MMR proteins listed above. Patients with this syndrome are at markedly increased risk for malignancies of the gastrointestinal (GI) tract (most commonly colon) and endometrium, less commonly also of the ovaries, upper urinary tract, and other organ systems. Among patients with dMMR/MSI-H solid tumors, 16% of these tumors are associated with LS [3]. dMMR/MSI-H status has been a topic of much research and discussion in the setting of the FDA’s recent tissue site-agnostic approval of pembrolizumab for advanced solid tumors demonstrating MSI-H/dMMR and without alternative treatment options after at least one line of therapy [6]. In a previous study out of Memorial Sloan Kettering (MSK) Cancer Center characterizing 15,045 cancer patients encompassing more than 50 cancer types, 4.1% of patients with MSI-H genomic signature carried a diagnosis of pancreatic cancer; in this same population, 14.7% of patients also carried diagnoses of LS [3].

In a randomized phase 3 trial of 307 individuals with previously untreated metastatic MSI-H/dMMR colorectal cancer (CRC), pembrolizumab was found to have led to significantly longer progression-free survival than chemotherapy (median: 16.5 versus 8.2 months; hazard ratio: 0.60; 95% confidence interval (CI): 0.45-0.80; P=0.0002), and evaluation of mature overall survival data is ongoing [15]. However, randomized phase 3 studies are ongoing evaluating first-line chemotherapy with or without immunotherapy and immunotherapy alone in MSI-H/dMMR metastatic colorectal cancer (chemotherapy plus atezolizumab, ClinicalTrials.gov number NCT02997228; nivolumab with or without ipilimumab, ClinicalTrials.gov number NCT04008030).

There remains a paucity of objective, large-scale data to define the role of immunotherapy in dMMR/MSI-H PDAC. In the absence of such studies, we rely on case reports of such experience and clinical trial data extrapolated from related disease types. Our case report adds to the growing body of literature emphasizing the therapeutic potential of immunotherapy in cases such as this. Regardless, larger datasets are needed, which will almost certainly require multi-institutional collaboration given the rarity of MMR deficiency and high MSI among patients with PDAC. In addition, there is a paucity of data in the literature with regard to the utilization of PD-1 alone or in combination with chemotherapy in the neoadjuvant setting in patients with resectable or borderline resectable PDAC. One question arises: should PD-1 alone be used? Moreover, in a patient who achieved radiographic CR prior to surgery, should surgery still be pursued? These questions can be better answered with more data.

## Conclusions

In conclusion, ctDNA can be invaluable in directing treatment decisions in a noninvasive, accessible manner, as was demonstrated in our case. Moreover, consideration should be given to immune-directed treatment approaches for dMMR/MSI-H PDAC, as there have been promising case reports of these agents for such patient populations. More research is needed.
